# Large Area Radial Junction Silicon Nanowire Solar Mini-Modules

**DOI:** 10.1038/s41598-018-20126-5

**Published:** 2018-01-26

**Authors:** Mutaz Al-Ghzaiwat, Martin Foldyna, Takashi Fuyuki, Wanghua Chen, Erik V. Johnson, Jacques Meot, Pere Roca i Cabarrocas

**Affiliations:** 1 LPICM, CNRS, Ecole Polytechnique, Université Paris-Saclay, 91128 Palaiseau, France; 2 SOLEMS, 3 rue Léon Blum, 91120 Palaiseau, France

## Abstract

In this work, we introduce the demonstration of 5 × 5 cm^2^ mini-modules based on radial junction silicon nanowire (RJ SiNW) devices grown by plasma-assisted vapor-liquid-solid (VLS) technique. The mini-modules are obtained thanks to an industrial laser scribing technique. The electrical parameters have been highlighted to address the performance of these devices and perspectives towards competitive RJ SiNW solar modules. Moreover, electroluminescence (EL) measurements were also conducted to assess the uniformity of the fabricated mini-modules. In addition, the structural characterization of solar cells and laser scribed lines has been assessed by scanning electron microscopy (SEM). The challenges and perspectives are also discussed.

## Introduction

Solar cells based on silicon thin film technology have been under development for years to achieve high energy conversion, low material consumption and low fabrication cost^1^. Recently, high energy conversion efficiency (η) of hydrogenated amorphous silicon (a-Si:H) (10.3%) and hydrogenated microcrystalline silicon (µc-Si:H) (11.77%) have been achieved^2,3^. However, the fabrication process requires additional steps and extra thick transparent conductive oxide (TCO) layers as compared to flat devices to obtain such high performance, such as improving light trapping and anti-reflection properties, which can be done by surface texturing and anti-reflection coating. Moreover, relatively thick absorber layers of a-Si:H (~300 nm) and µc-Si:H (~2 µm) have been used. On the other hand, a promising research field based on radial junction silicon nanowires (RJ SiNWs) provides several advantages over the traditional planar junction solar cells, including high built-in electrical field due to the ultra-thin absorber layer (~100 nm), enhanced light trapping and anti-reflection properties. In addition, the collection of photo-generated carriers is decoupled due to the unique geometry of NWs^4,5^.

Recently, an efficiency of ~9.2% of SiNW solar cells for 0.126 cm^2^ area has been reached^6^. Nevertheless, upscaling and compatibility with industrial processes are required to move PIN RJ devices to large area production. However, increasing the active area of PIN devices may reduce the open-circuit voltage (V_oc_), fill factor (FF) and thus limit the device performance. Therefore, for practical reasons, the large area of solar devices is separated into small active segments (cells) with an assistance of mechanical or laser scribing techniques^7–9^, to provide a monolithical integration of isolated solar cells connected in series. Laser scribing (ablation) provides advantages over mechanical scribing in terms of less mechanical stress on the devices, shorter processing time and higher reliability. Moreover, laser scribing is a non-contact process with a selective and precise removal of thin film materials by focused laser beam.

The integration of solar cells into modules using laser scribing was firstly granted to J. J. Hanak in 1981^10^. Since then, laser scribing has been employed for different type of solar cells including a-Si:H, µc-Si:H^11,12^ and CIGS^13,14^ devices, by using various types of lasers^15^. Typically, for each thin film material, laser scribing parameters, such as the wavelength and pulse duration, have to be optimized to reduce the creation of flakes and re-solidified material^16^, which may cause short circuits in the final devices^13^. Several research groups have provided different approaches to avoid the insufficient removal of electrodes and thin film materials^7,15,17^. Performing laser scribing on the solar devices creates a dead area known also as lost area. The presence of the dead area will slightly reduce the active area, but it is more than satisfactorily compensated by the performance of smaller devices in series. However, the dead area can be minimized as demonstrated by Hass *et al*. with a new concept of series connection of individual cells, replacing the typical stripe-like scribing, by rearranging the laser spots^18^. Other approaches mainly based on optimizing laser parameters can be found elsewhere^19^.

In this paper, we demonstrate for the first time, the fabrication of mini-modules (active area of 10 cm^2^) based on RJ architecture. The fabrication is based on PECVD NW growth and material deposition combined with laser scribing technique for proper modularization. Section 2 describes samples, laser scribing setups and characterization techniques applied to fabricated solar mini-modules. Results in Section 3 are split into three subsections devoted to RJ deposition, followed by the description of light scribing and mini-module fabrication. The last subsection is devoted to the characterization of the device performance and the discussion on week and strong points of presented solar mini-modules. The conclusions are presented in Section 4.

## Samples description and characterization techniques

In this work, we have used 5 × 5 cm^2^ commercial substrates having 600 nm of fluorine doped tin oxide (FTO), deposited on 2.2 mm thick soda lime glass using chemical vapor deposition (CVD) technique. The role of FTO is to provide back electrode in the fabricated SiNW mini-modules. The surface of FTO has been reduced using hydrogen (H_2_) plasma at 200 °C to form an elemental Sn^20,21^. The preparation of Sn catalyst is a crucial step to initiate the growth of SiNWs using plasma assisted VLS technique^5,22,23^. Moreover, Sn has several advantages including low melting temperature (232 °C) which is suitable for low temperature PECVD reactors, abundancy, non-toxicity^24^, low cost related to its abundance and it does not introduce deep electronic defects in Si as compared to gold^4,25^. The FTO layer reduces the fabrication process steps by providing the electrode and Sn catalyst simultaneously, rather than the typical process demonstrated by Misra *et al*. which requires additional step of Sn catalyst evaporation^26^.

The n-type window layer of the fabricated PIN RJ SiNW mini-modules has been deposited using another PECVD reactor. We have deposited a hydrogenated microcrystalline silicon oxide (µc-SiO_x_:H) layer which is a phase mixture of µc-Si:H and hydrogenated amorphous silicon oxide (a-SiO_x_:H)^27,28^. The n-doping mainly affects the µc-Si:H phases. The n-type µc-Si:H provides the channel for sufficient electrical conductivity, while the a-SiO_x_:H phase insures a high optical transparency and low refractive index^29^. Therefore, the absorption in the window layer will be minimized leading to improve device performances^2,6^.

To finalize the mini-modules, two different laser scribing apparatus with different lasers have been used. The first one is based on YAG (1.064 µm) nanosecond pulsed laser with Q-switch supplying short pulses of power, suitable to scribe the (TCO) thin films. The other setup has YAG (532 nm) nanosecond pulsed laser with Q-switch for silicon material scribing. After fabricating the SiNW mini-modules, 2 isolation scribes obtained by laser have been made close to edges of the substrate. This eliminates undesired possible short-circuiting at the borders of mini-module.

The area of the top electrode of these mini-modules was defined using lift off technique. It started with paste of titanium dioxide (TiO_2_) spread on the surface of the PIN RJs in selected lines, followed by sputtering of TCO layer. Finally, the paste was removed by rinsing the samples in alcohol.

To evaluate the density and morphology of SiNWs, as well as the scribes made on the substrates, SEM (HITACHI S-4800) has been utilized. In addition, energy-dispersive X-ray microscopy (EDX) has been used, to analyze the elemental components of targeted areas of SiNW mini-modules. The EDX (UltraDry EDX Detector) microanalysis system was provided by Thermo Scientific and it is integrated in SEM system.

To study the electrical performance of the fabricated SiNW mini-modules, current density-voltage (J-V) characteristics have been recorded under standard AM1.5 G illumination conditions using Newport solar simulator (class AAA certified)^30^. To obtain the efficiency of a solar device, equation (1) has been used. The output electrical power (the multiplication of V_oc_, short circuit current (I_sc)_ and FF) is divided by the input solar power (P_in_, equivalent to 1 kW/m^2^ multiplied by the active area of the measured solar device). Moreover, EL characterization was carried out on the fabricated SiNW mini-modules using a homemade setup^31,32^. After applying forward bias voltage through connected probes, the light emission, due to radiative recombination of carriers is captured by a charge coupled device (CCD) camera. The CCD camera has a silicon detector with a bandpass filter removing wavelengths below 900 nm. In addition, the presence of series resistance of top TCO has been confirmed by this technique^33,34^.

1 $${\boldsymbol{\eta }}=\frac{{\boldsymbol{Voc}}.{\boldsymbol{Isc}}.{\boldsymbol{FF}}}{{\boldsymbol{Pin}}.{\boldsymbol{Area}}}$$

## Results and Discussion

### Silicon nanowire growth and PIN radial junction deposition

Silicon NWs were grown on the top of 25 cm^2^ FTO substrates by plasma assisted VLS growth process. After loading the sample inside the PECVD reactor, H_2_ plasma was ignited for 2 minutes at RF power of 5 W, the H_2_ pressure was fixed at 600 mTorr at flow rate of 100 standard cubic centimeters per minute (sccm) and the substrate temperature was 200 °C. We have used plasma conditions for which the sheet resistance increases from ~10 to ~12 Ω/□ (estimated measurement error is below 1 Ω/□), which is still acceptable.

**Figure 1:**
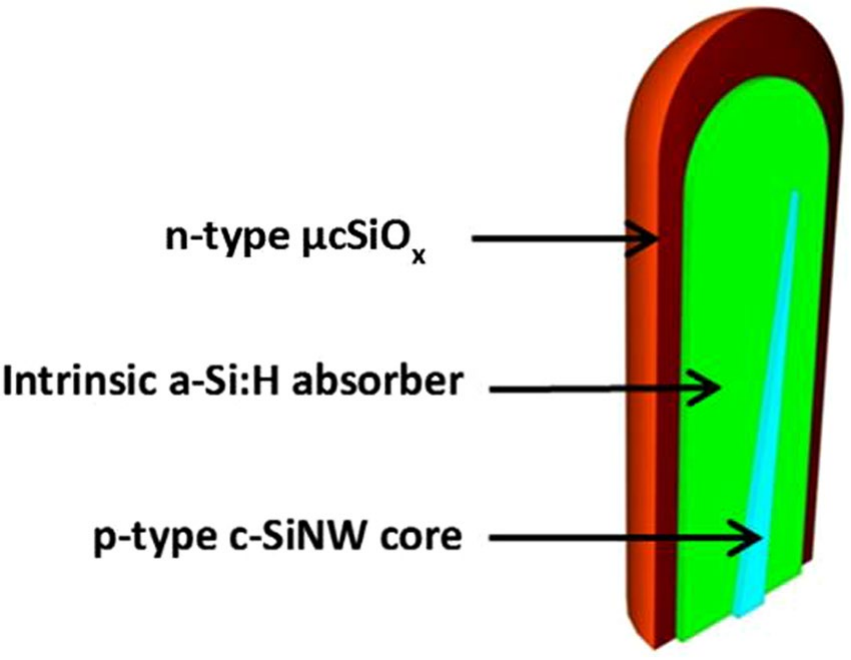
Schematic showing the plasma assisted VLS grown p-type SiNW, coated with 100 nm intrinsic a-Si:H absorber layer and n-type µc-SiOx:H layer.

To initiate the growth of SiNWs, the substrate temperature was increased to 400 °C, and a mix of 3 sccm SiH_4_, 100 sccm H_2_ and 1 sccm trimethylboron (TMB, 1% in SiH_4_) was introduced at pressure of 1 Torr, the RF power was 2 W for 10 minutes. The length of the self-assembled grown p-type SiNWs is around 0.6–0.8 µm with the top and bottom diameters being ~20 and ~50 nm, respectively. Subsequently, the temperature was decreased to 180 °C, and an intrinsic a-Si:H absorber layer was deposited with 3 sccm of SiH_4_ at 120 mTorr and RF power of 0.9 W. The thickness of the absorber layer was around 100 nm after 60 min of deposition. Afterwards, n-type µc-SiO_x_:H was deposited on the top of the absorber layer in the presence of SiH_4_, H_2_, PH_3_ (1% in H_2_) and CO_2_ at chamber pressure of 2.4 Torr. The plasma power and substrate temperature were 16 W and 150 °C, respectively. Additional details regarding the fabrication process can be found elsewhere^26^. The final structure of the grown individual RJ SiNW is shown in Fig. 1.

### Laser scribing and mini-modules fabrication

To build mini-modules based on RJ SiNWs, 25 cm^2^ substrates of 600 nm FTO on 2.2 mm thick glass have been used. Typically, laser scribing process consists of three main steps to split the large cell into individual stripes: (i) P1, a selective laser scribing of the back contact to insure electrical isolation between the separated TCO segments. (ii) P2, a selective laser scribing of PIN junction material to connect the top and back contacts. (iii) P3, a selective deposition of the top contact followed by lift off technique to insure isolation of each individual solar cell.

**Figure 2:**
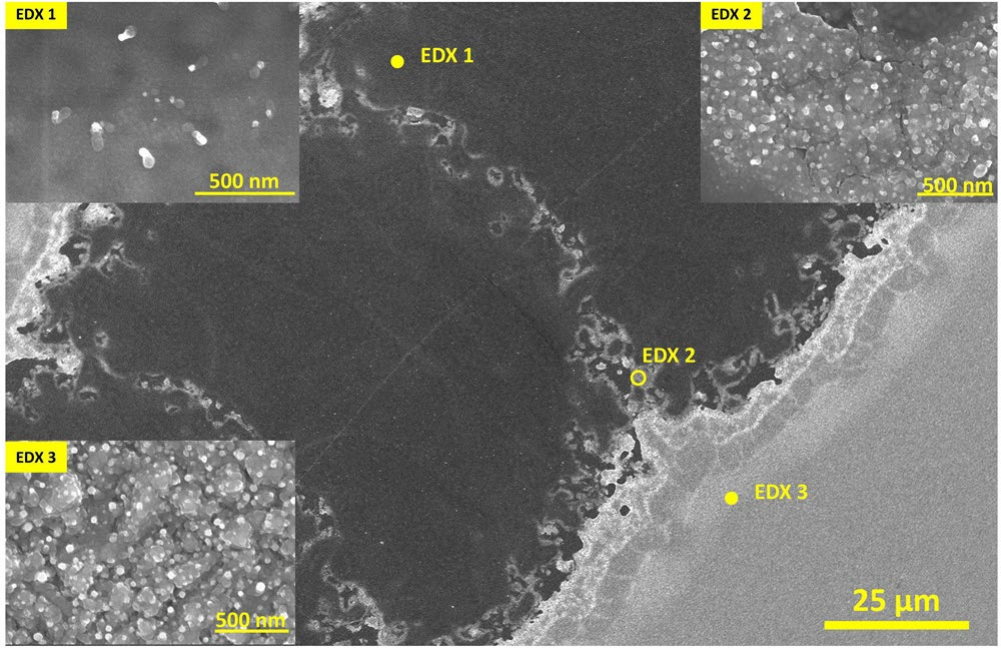
SEM micrograph of P1 scribe (on FTO/glass substrates) after performing H_2_ treatment and depositing p-type a-Si:H at 250 ˚C. The insets present three positions showing the features of the deposited p-type a-Si:H. (EDX 1) the scribed area of FTO with less SiNWs. (EDX 2) Many SiNWs caused by growing on residual FTO between two separated laser pulses. (EDX 3) The deposited p-type a-Si:H on the top of FTO.

For P1, prior to loading the substrates into the PECVD reactor, laser scribing using YAG (1.064 µm) was conducted on the FTO back contact layer as shown in Fig. 2. The scribing width on the FTO layer was around 120 µm. The uniformity of FTO removal indicates the accuracy of laser beam intensity.

**Figure 3:**
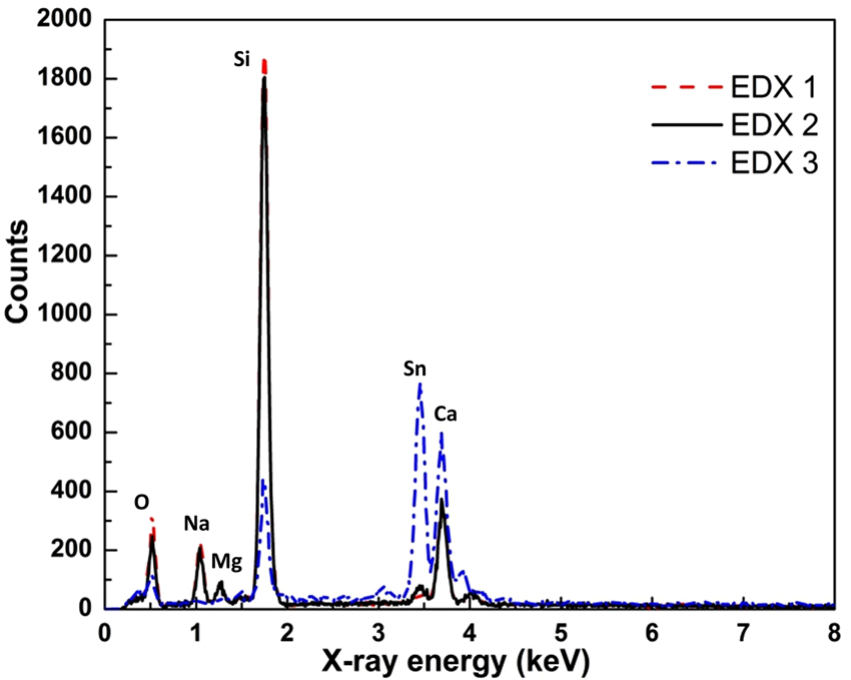
EDX analysis on different positions after depositing p-type a-Si:H. The black peak at 3.44 keV identifies the Sn presence within the scribed area.

A study was carried out on P1 scribe to analyze the presence of Sn within the scribed area, which may cause undesired shunting of the final mini-modules. We performed a H_2_ plasma treatment followed by the deposition of p-type a-Si:H for 5 minutes at 250 °C, which can lead to a SiNW growth in the presence of metallic Sn. We have indeed observed the growth of SiNWs on the samples in areas with residual FTO. To determine the material composition, we performed EDX analysis on three positions on the sample shown in Fig. 2, which are presented in the insets of Fig. 2. Position EDX 1 is in the area with better scribing, EDX 2 in the area occupied by many NWs and the position EDX 3 in the area with indium tin oxide (ITO) for the reference. In EDX 1, we still can observe few SiNWs within the previously ablated area. The SiNWs grown in the insufficiently scribed area (EDX 2) have similar features as the ones grown directly on the non-scribed area (EDX 3). All three positions were analyzed using EDX and their comparison of spectra as shown in Fig. 3. The main elements of soda lime glass, which are Si, O, Mg, Ca and Na, were detected in the EDX analysis. The largest peak within P1 scribe is for Si. Moreover, the Sn peak at 3.44 keV is more pronounced in the spectrum taken at the position EDX 2 as compared to position EDX 1. This means that the removal of FTO was not complete and it explains the growth of SiNWs in those areas. The measured resistance, between the separated segments, after the deposition of a-Si:H has been decreased from over 500 to ~300 MΩ. The small presence of Sn within the scribed area may contribute in electrical shunting, and can limit the performance of SiNW mini-modules.

**Figure 4:**
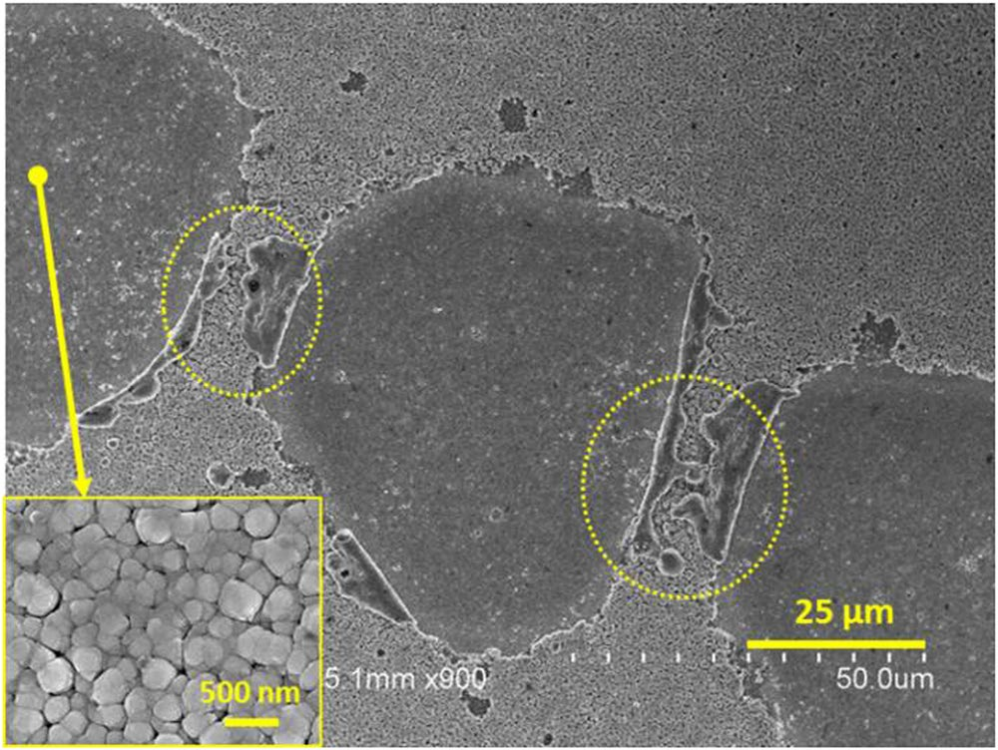
SEM micrograph on P2 scribe after obtaining the SiNW mini-module. The yellow dashed circles show the re-solidified Si material after applying the laser. Inset shows tilted SEM micrograph of residual material and SiNWs after P2 laser scribing.

Second scribing step (P2) is a selective laser ablation of PIN junction material connects the top and back contacts. After depositing the PIN RJs (using the previously discussed process), the substrates were scribed using YAG (532 nm) nanosecond pulsed laser with Q-switch. Laser spots on PIN RJ layer have a width ~ 70 µm as shown in Fig. 4. Re-solidified Si material can be clearly observed at the borders of the scribed area. The size of each spot is modified by the diffusion of the laser beam on the surface of the SiNWs during the scribing process. The inset in Fig. 4 shows that not all RJs have been removed after P2 laser scribing, suggesting that removal of SiNWs requires different conditions than planar junction thin films.

**Figure 5:**
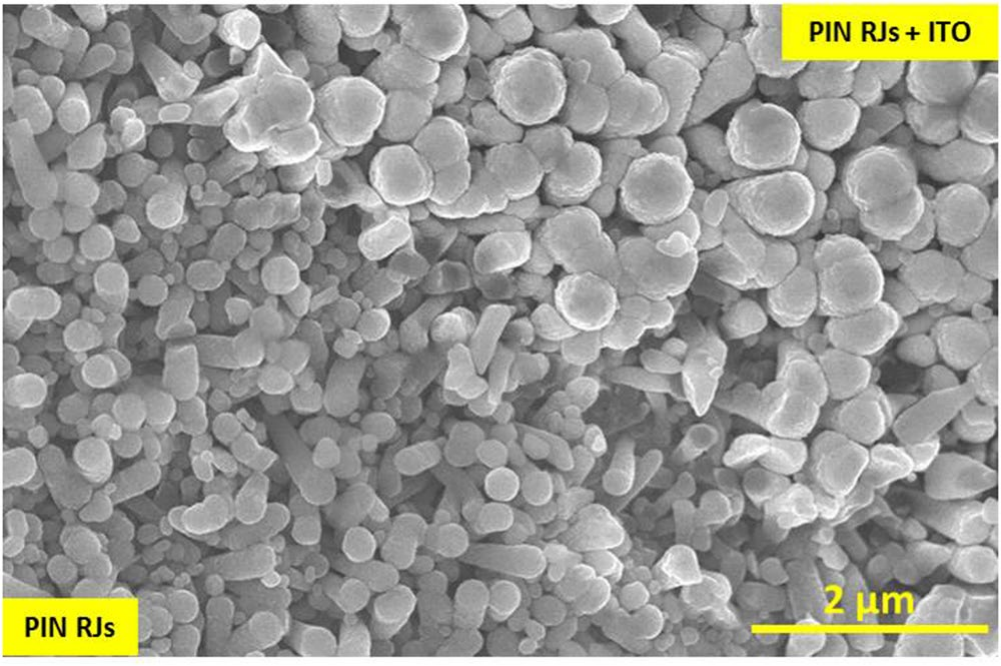
Tilted SEM micrograph showing the ITO sputtered on top of SiNWs along with the uncovered SiNWs. The sputtering on SiNWs was followed by lift off technique.

**Figure 6:**
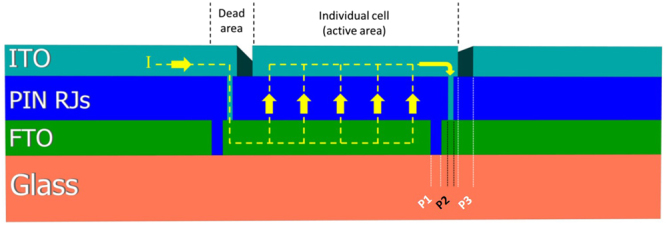
Schematics showing the geometry of laser scribed mini-modules. The yellow dashed line illustrates the electrical current path through the interconnected cells.

An ITO was sputtered with nominal thickness of 300 nm (measured on the flat surface) on top of RJ SiNWs. The area of the ITO contacts was defined using lift off technique. At first, the paste of TiO_2_ was spread on PIN RJs with 0.45 mm broad lines, which was followed by the deposition of ITO on the top of the PIN RJ, shown in Fig. 5. The top diameters of SiNWs after the sputtering of ITO are ~500 nm, in contrast to the top diameters of RJs in regions without ITO which are 300 nm. It shows that ITO sputtering is not conformal and only up to 100 nm is deposited on the RJ walls. Structure of all processed layers (including FTO, RJs and ITO) after applying laser scribing and deposition of SiNWs is schematically presented in Fig. 6. The photo-generated current will flow (in series) through the RJ layer from one segment to another leading to cumulated voltage at the external electrodes of the mini-module.

A reference PIN solar cell has been fabricated to compare its performance with that of the fabricated mini-modules. We have used the same substrates (5 × 5 cm^2^) without using P2 and P3 steps and instead, 280 nm thick ITO was sputtered on the top of PIN RJs through a shadow mask with circular pads of 2 and 4 mm diameters. Prior the characterization, all samples have been annealed for 1 hour at 180 °C in air.

### Performance of RJ SiNW mini-modules

**Figure 7:**
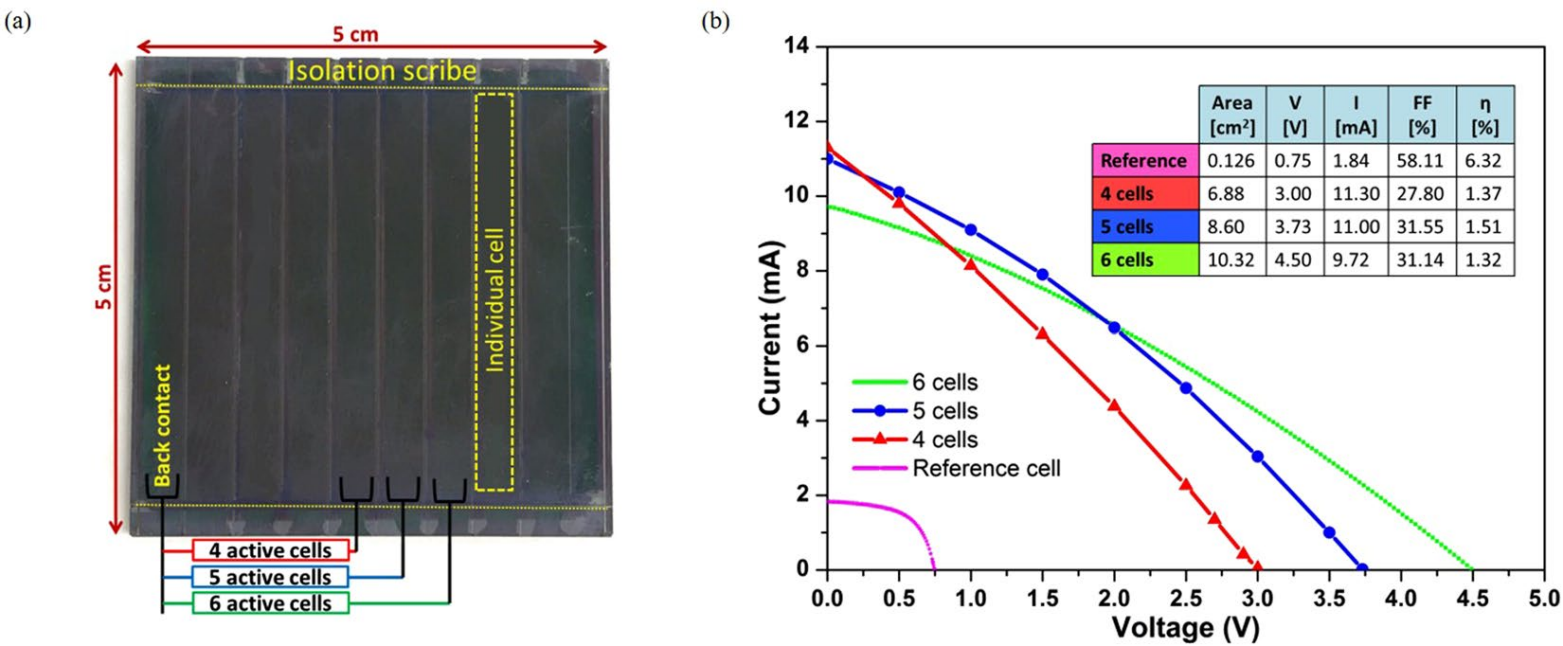
(**a**) RJ SiNW solar mini-module fabricated by PECVD using plasma-assisted VLS technique, with assistance of laser scribing for cells separation. (**b**) I-V characteristics of mini-module based on RJ SiNWs of 4, 5 and 6 interconnected cells in comparison with a reference SiNW solar cell (pink curve). The electrical parameters are given in the inset table in the graph.

The photo of SiNW solar mini-module fabricated with the assistance of the laser scribing technique is presented in Fig. 7(a). These mini-modules have 10 active cells connected electrically in series. In order to measure I-V characteristics of a group of RJ SiNW cells, we select area of one cell to act as a back contact and we select n + 1^th^ cell area as a front contact. E.g. for 4, 5 and 6 cells groups we took front contact on 4^th^, 5^th^ and 6^th^ cells next to our back contact cell area [see Fig. 7(a)]. The Ag contacts were painted using liquid Ag paste on the surface of the front and back contact cells, to improve the photo-generated current collection and to reduce the impact of the lateral series resistance of ITO. Note that full metallic grid is planned for the future with adequate design for efficient collection.

The electrical performance of the fabricated SiNW solar mini-modules is shown in the Fig. 7(b). The active area of the mini-module is defined by 4, 5 and 6 interconnected cells with dimensions of 4.3 × 0.4 cm^2^, with total active area of 6.88, 8.60 and 10.32, respectively. After measuring I-V under the solar simulator, the results show a high V_oc_ of 3.00, 3.73 and 4.50 V (~0.75 V per cell), respectively. This indicates a satisfactory V_oc_ of the individual cells. In addition, the V_oc_ for each cell is in a good agreement with the reference sample that has been discussed previously. A high I_sc_ of 11.30, 11.00 and 9.72 mA in individual cell have been obtained by 4, 5 and 6 RJ SiNW cells compared to 1.84 mA of the reference cell. However, in terms of short circuit current density (J_sc_) which takes in consideration the active area of the cells in the calculations, the values are smaller for mini-module compared to J_sc_ of 14.65 mA/cm^2^ measured on the reference cell. The mini-module cells provide J_sc_ of 6.57, 6.4 and 5.65 mA/cm^2^ in individual cell of 4, 5 and 6 cells, respectively. In 4 cells connection, the I_sc_ increased by ~16% compared to 6 cells.

**Figure 8:**
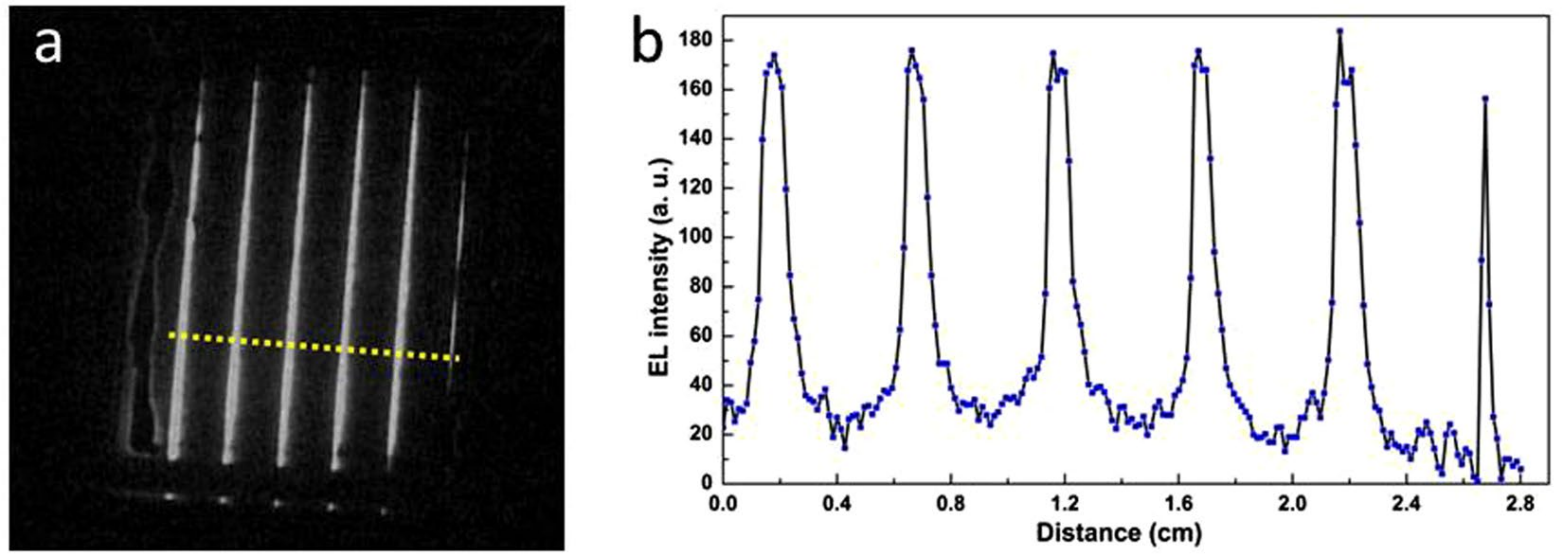
(**a**) EL image obtained after removing the background light using ImageJ software. (**b**) EL profile along the yellow dashed line. The lowering of EL intensity away from the injecting part indicates high resistive losses due to ITO.

Nevertheless, these mini-modules still have lower FF as compared to the reference cell and reduced η. This is maybe due to a high series resistance of these devices originating mainly from the insufficient removal of PIN material. The presence of series resistance in the top contact (TCO) was inspected by EL technique. Figure 8(a) shows EL image of RJ SiNW mini-module obtained by CCD camera, taken with an applied voltage of 13 V. Figure 8(b) shows the EL intensity plotted along the horizontal distance of the interconnected cells [yellow dashed line in Fig. 8(a)]. At the injection edge of each individual cell, a high EL emission can be observed. However, the EL emission decreases very fast at the collection edge which is mainly due to the high resistive losses in the top ITO contact.

Even though there are many challenges associated with scribing, as detailed in Section 2, the SiNW mini-module exhibit good V_oc_. The V_oc_ of 4.5 V has been achieved on the large active area of about 10 cm^2^. In addition, the achieved power generation of 13 mW for PECVD RJ SiNW cells is the first to our knowledge up to date. Demonstrated mini-module shows a clear potential for transfer of the nanowire radial junction technology to larger scale with compatibility with existing thin film industry due to the use of well-established PECVD and laser scribing techniques.

## Conclusion

For the first time, a successful monolithic interconnection of 6 individual PIN RJ SiNW cells has been realized, with active area of 10.32 cm^2^. A high V_oc_ of 4.5 V has been obtained, precisely six times higher than the reference cell, leading to a power generation of 13 mW. These results provide a new strategy to push forward the device performance of RJ SiNW solar modules, while keeping the compatibility with well-established industrial techniques. This work demonstrates a synergy between scientifically interesting nanowire based solar cells and requirements of a low cost and large surface area for industrial production.
